# 3,3′-Dibenzoyl-1,1′-(3,6-dioxaoctane-1,8-di­yl)dithio­urea

**DOI:** 10.1107/S1600536809005662

**Published:** 2009-02-21

**Authors:** Mouhamadou Moustapha Sow, Ousmane Diouf, Aliou Hamady Barry, Mohamed Gaye, Abdou Salam Sall

**Affiliations:** a Département de Chimie, Faculté des Sciences et Techniques, Université Cheikh Anta Diop, Dakar, Senegal; b Département de Chimie, Faculté des Sciences, Université de Nouakchott, Nouakchott, Mauritania

## Abstract

In the mol­ecule of the title compound, C_22_H_26_N_4_O_4_S_2_, the central O—CH_2_—CH_2_—O chain adopts a synclinal conformation [torsion angle = 65.0 (2)°]. The crystal structure is stabilized by intra­molecular N—H⋯O=C and inter­molecular N—H⋯O—C hydrogen bonds.

## Related literature

For related structures, see: Avşar *et al.* (2003 ▶); Arslan *et al.* (2004 ▶); Du & Du (2008 ▶); Ding *et al.* (2008 ▶).
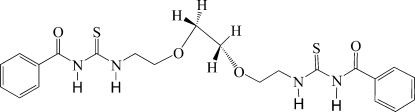


## Experimental

### 

#### Crystal data

C_22_H_26_N_4_O_4_S_2_
*M* *_r_* = 474.59
Triclinic, 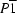

*a* = 7.9718 (2) Å
*b* = 9.2177 (3) Å
*c* = 16.4106 (5) Å
α = 81.018 (2)°
β = 83.364 (2)°
γ = 80.450 (2)°
*V* = 1169.60 (6) Å^3^
*Z* = 2
Mo *K*α radiation
μ = 0.26 mm^−1^
*T* = 293 K
0.10 × 0.10 × 0.10 mm

#### Data collection

Nonius Kappa CCD diffractometer
Absorption correction: none
7989 measured reflections
4211 independent reflections
2737 reflections with *I* > 2σ(*I*)
*R*_int_ = 0.030

#### Refinement

*R*[*F* ^2^ > 2σ(*F* ^2^)] = 0.045
*wR*(*F* ^2^) = 0.128
*S* = 1.02
4211 reflections
305 parameters
H atoms treated by a mixture of independent and constrained refinement
Δρ_max_ = 0.41 e Å^−3^
Δρ_min_ = −0.50 e Å^−3^

### 

Data collection: *COLLECT* (Nonius, 1998 ▶); cell refinement: *DENZO* and *SCALEPACK* (Otwinowski & Minor, 1997 ▶); data reduction: *DENZO* and *SCALEPACK*; program(s) used to solve structure: *SHELXS97* (Sheldrick, 2008 ▶); program(s) used to refine structure: *SHELXL97* (Sheldrick, 2008 ▶); molecular graphics: *PLATON* (Spek, 2009 ▶); software used to prepare material for publication: *SHELXL97*.

## Supplementary Material

Crystal structure: contains datablocks I, global. DOI: 10.1107/S1600536809005662/bh2217sup1.cif

Structure factors: contains datablocks I. DOI: 10.1107/S1600536809005662/bh2217Isup2.hkl

Additional supplementary materials:  crystallographic information; 3D view; checkCIF report

## Figures and Tables

**Table 1:** Hydrogen-bond geometry (Å, °)

*D*—H⋯*A*	*D*—H	H⋯*A*	*D*⋯*A*	*D*—H⋯*A*
N2—H2⋯O1	0.82 (3)	1.99 (3)	2.648 (3)	136 (2)
N3—H3⋯O4	0.83 (3)	2.01 (3)	2.632 (3)	132 (3)
N4—H4⋯O2^i^	0.82 (3)	2.48 (3)	3.290 (3)	170 (2)

Symmetry code: (i) 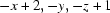.

**Fig. 1.**
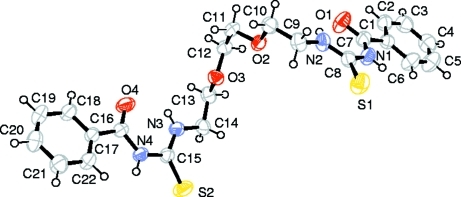
An ORTEP view of the asymmetric unit of the title compound, showing the atom-numbering scheme. Displacement ellipsoids are plotted at the 50% probability level.

**Fig. 2.**
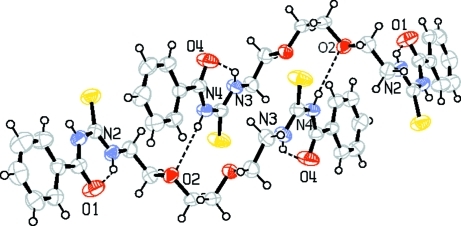
Molecular representation of the compound showing hydrogen bonds (dashed lines).

## supplementary crystallographic information

### Comment

The title compound, C_22_H_26_N_4_O_4_S_2_, was characterized by ^1^H and ^13^C NMR, solid-state IR and X-ray crystallographic techniques. The X-ray structure determination reveals that the compound crystallizes in the triclinic space group *P*1 with one molecule in the asymmetric unit. The molecular geometry is illustrated in Fig. 1. The C—S bond lengths of 1.665 (3) Å and 1.659 (2) Å and the C—O bond lengths of 1.220 (3) Å and 1.222 (3) Å are double bonds character and are comparable to those observed for 1-(biphenyl-4-carbonyl)-3-*p*-tolyl-thiourea [1.647 (3) Å for C—S, 1.217 (3) and 1.224 (3) Å for C—O respectively (Arslan *et al.*, 2004)]. The C—N bond lengths are in the range 1.310 (3)–1.451 (3) Å, and are shorter than the normal single C—N bond length (Avşar *et al.*, 2003). The carbonyl group forms an intramolecular hydrogen bonds with the N2—H2 and the N3—H3 groups, which forms two six-membered rings (C8/N1/C7/O1/H2/N2 and C15/N4/C16/O4/H3/N3) structure (Fig. 2); H2···O1 and the H3···O4 separations are respectively 1.99 (3) Å and 2.01 (3) Å. There is an intermolecular hydrogen bonding between N4—H4 and the O atom of the ethoxy group of a symmetry-related molecule, the H4···O2 (-*x* + 2, -*y*, -*z* + 1) separation being 2.48 (3) Å (Table 1). The structure of the title compound is related to other thiourea derivatives (*e.g*. Ding *et al.*, 2008; Du & Du, 2008).

### Experimental

Benzoyl chloride (7.10 g, 50 mmol) was reacted with potassium thiocyanate (4.86 g, 50 mmol) in CH_3_OCH_3_ (50 ml) solution, to give the corresponding benzoyl isothiocyanate after one hour under refluxing. After cooling to room temperature, a solution of 2-(2-(2-aminoethoxy)ethoxy)ethanamine (3.70 g, 25 mmol) in CH_3_OCH_3_ (20 ml) was added dropwise to benzoyl isothiocyanate. After three hours under stirring, 200 ml of HCl 1 *M* was added. A yellow oil was isolated and treated with diethyl ether to give the title compound which is washed with diethyl ether twice. Yield: 55.9%. m.p. 415–419 K. Anal. Calc. for C_22_H_26_N_4_O_4_S_2_: C 55.68, H 5.52, N 11.81%. Found: C 55.70, H 5.45, N 11.65%. Selected IR data (cm^-1^, KBr pellet): 3424, 3218 (ν NH), 1667 (ν C?O), 1160 (ν C?S). ^1^H-NMR (200 MHz, DMSO-d_6_, δ, p.p.m.): 3.45 (t, 4H, N—CH_2_); 3.63 (s, 4H, O—CH_2_); 3.70 (t, 4H, O—CH_2_); 7.21–7.92 (m, 10H, C_6_H_5_); 11.01 (s, 2H, NH); 11.41 (s, 2H, NH). ^13^C-NMR (50 MHz, DMSO-d_6_, δ, p.p.m.): 45.21 (N—CH_2_); 68.09 (O—CH_2_); 70.12 (O—CH_2_); 133.31–127.62 (C_6_H_5_); 1168.60 (C?O); 180.81 (C?S). A CH_3_Cl solution of the title compound was mixed with ethanol (1/1). After several days, colorless block-shaped single crystals suitable for X-ray crystallographic analysis were obtained.

### Refinement

H atoms of NH groups were located in a difference map and refined freely. Others H atoms were placed geometrically and refined with a riding model. *U*_iso_(H) for H was calculated as 1.2 *U*_eq_ of the carrier atom.

### Crystal data

**Table:**

C_22_H_26_N_4_O_4_S_2_	*Z* = 2
*M**_r_* = 474.59	*F*(000) = 500
Triclinic, *P*1	*D*_x_ = 1.347 Mg m^−^^3^
Hall symbol: -P 1	Melting point: 415 K
*a* = 7.9718 (2) Å	Mo *K*α radiation, λ = 0.71073 Å
*b* = 9.2177 (3) Å	Cell parameters from 4206 reflections
*c* = 16.4106 (5) Å	θ = 1.0–25.4°
α = 81.018 (2)°	µ = 0.26 mm^−^^1^
β = 83.364 (2)°	*T* = 293 K
γ = 80.450 (2)°	Prism, yellow
*V* = 1169.60 (6) Å^3^	0.10 × 0.10 × 0.10 mm

### Data collection

**Table:**

Nonius KappaCCD diffractometer	2737 reflections with *I* > 2σ(*I*)
Radiation source: fine-focus sealed tube	*R*_int_ = 0.030
graphite	θ_max_ = 25.2°, θ_min_ = 2.3°
ω scans	*h* = −9→9
7989 measured reflections	*k* = −11→11
4211 independent reflections	*l* = −19→19

### Refinement

**Table:**

Refinement on *F*^2^	Primary atom site location: structure-invariant direct methods
Least-squares matrix: full	Secondary atom site location: difference Fourier map
*R*[*F*^2^ > 2σ(*F*^2^)] = 0.045	Hydrogen site location: inferred from neighbouring sites
*wR*(*F*^2^) = 0.128	H atoms treated by a mixture of independent and constrained refinement
*S* = 1.02	*w* = 1/[σ^2^(*F*_o_^2^) + (0.0614*P*)^2^ + 0.2609*P*] where *P* = (*F*_o_^2^ + 2*F*_c_^2^)/3
4211 reflections	(Δ/σ)_max_ = 0.008
305 parameters	Δρ_max_ = 0.41 e Å^−^^3^
0 restraints	Δρ_min_ = −0.50 e Å^−^^3^
0 constraints	

### Fractional atomic coordinates and isotropic or equivalent isotropic displacement parameters (Å^2^)

**Table:**

	*x*	*y*	*z*	*U*_iso_*/*U*_eq_	
S1	0.37094 (10)	0.16706 (9)	0.86251 (5)	0.0713 (3)	
S2	1.26452 (13)	−0.27342 (8)	0.52516 (4)	0.0790 (3)	
O1	0.7377 (3)	0.4586 (2)	0.91685 (11)	0.0711 (6)	
O2	0.81088 (19)	0.39803 (17)	0.67050 (9)	0.0482 (4)	
O3	1.12041 (19)	0.24744 (17)	0.60508 (10)	0.0472 (4)	
O4	1.2463 (3)	0.1608 (2)	0.35665 (11)	0.0740 (6)	
N1	0.5641 (3)	0.2839 (3)	0.94678 (14)	0.0527 (6)	
H1	0.530 (3)	0.224 (3)	0.9815 (16)	0.049 (8)*	
N2	0.5680 (3)	0.3729 (2)	0.80784 (13)	0.0492 (5)	
H2	0.639 (3)	0.422 (3)	0.8172 (16)	0.056 (8)*	
N3	1.2498 (3)	0.0165 (3)	0.50822 (13)	0.0506 (5)	
H3	1.236 (3)	0.099 (3)	0.4798 (17)	0.063 (9)*	
N4	1.2073 (3)	−0.0786 (2)	0.39123 (12)	0.0465 (5)	
H4	1.190 (3)	−0.154 (3)	0.3741 (15)	0.052 (8)*	
C1	0.7116 (3)	0.3433 (3)	1.05616 (14)	0.0456 (6)	
C2	0.8101 (3)	0.4370 (3)	1.08034 (16)	0.0539 (6)	
H2A	0.8497	0.5115	1.0416	0.065*	
C3	0.8498 (4)	0.4211 (3)	1.16088 (18)	0.0639 (7)	
H3A	0.9169	0.4840	1.1763	0.077*	
C4	0.7902 (4)	0.3120 (3)	1.21864 (18)	0.0692 (8)	
H4A	0.8165	0.3012	1.2732	0.083*	
C5	0.6923 (4)	0.2194 (4)	1.19573 (19)	0.0771 (9)	
H5	0.6513	0.1464	1.2350	0.093*	
C6	0.6537 (4)	0.2335 (3)	1.11472 (17)	0.0649 (8)	
H6	0.5885	0.1690	1.0995	0.078*	
C7	0.6744 (3)	0.3674 (3)	0.96796 (15)	0.0488 (6)	
C8	0.5078 (3)	0.2817 (3)	0.86956 (15)	0.0477 (6)	
C9	0.5275 (3)	0.3838 (3)	0.72324 (15)	0.0537 (6)	
H9A	0.4095	0.4293	0.7189	0.064*	
H9B	0.5406	0.2851	0.7075	0.064*	
C10	0.6419 (3)	0.4745 (3)	0.66582 (15)	0.0516 (6)	
H10A	0.6101	0.4859	0.6096	0.062*	
H10B	0.6331	0.5723	0.6822	0.062*	
C11	0.9346 (3)	0.4733 (3)	0.61860 (16)	0.0522 (6)	
H11A	0.9278	0.5730	0.6320	0.063*	
H11B	0.9133	0.4804	0.5610	0.063*	
C12	1.1067 (3)	0.3882 (3)	0.63208 (16)	0.0506 (6)	
H12A	1.1936	0.4426	0.6014	0.061*	
H12B	1.1243	0.3751	0.6904	0.061*	
C13	1.2832 (3)	0.1611 (3)	0.61572 (16)	0.0529 (6)	
H13A	1.3092	0.1538	0.6727	0.063*	
H13B	1.3705	0.2077	0.5799	0.063*	
C14	1.2803 (3)	0.0091 (3)	0.59436 (14)	0.0504 (6)	
H14A	1.3887	−0.0529	0.6045	0.061*	
H14B	1.1912	−0.0359	0.6297	0.061*	
C15	1.2389 (3)	−0.1016 (3)	0.47501 (14)	0.0447 (6)	
C16	1.2118 (3)	0.0483 (3)	0.33592 (14)	0.0454 (6)	
C17	1.1796 (3)	0.0433 (2)	0.24907 (14)	0.0419 (5)	
C18	1.2227 (3)	0.1595 (3)	0.19018 (15)	0.0527 (6)	
H18	1.2684	0.2361	0.2062	0.063*	
C19	1.1984 (3)	0.1624 (3)	0.10851 (16)	0.0610 (7)	
H19	1.2299	0.2398	0.0693	0.073*	
C20	1.1281 (3)	0.0520 (3)	0.08436 (16)	0.0618 (7)	
H20	1.1120	0.0546	0.0289	0.074*	
C21	1.0811 (3)	−0.0632 (3)	0.14224 (16)	0.0605 (7)	
H21	1.0324	−0.1378	0.1260	0.073*	
C22	1.1069 (3)	−0.0672 (3)	0.22467 (15)	0.0507 (6)	
H22	1.0751	−0.1446	0.2638	0.061*	

### Atomic displacement parameters (Å^2^)

**Table:**

	*U*^11^	*U*^22^	*U*^33^	*U*^12^	*U*^13^	*U*^23^
S1	0.0807 (5)	0.0781 (5)	0.0659 (5)	−0.0445 (4)	−0.0032 (4)	−0.0115 (4)
S2	0.1467 (8)	0.0464 (4)	0.0494 (4)	−0.0296 (5)	−0.0275 (5)	0.0059 (3)
O1	0.0906 (14)	0.0822 (14)	0.0503 (11)	−0.0502 (12)	−0.0123 (10)	0.0050 (10)
O2	0.0498 (10)	0.0443 (9)	0.0461 (9)	−0.0077 (8)	0.0014 (8)	0.0036 (7)
O3	0.0497 (10)	0.0457 (9)	0.0501 (10)	−0.0119 (8)	−0.0053 (7)	−0.0140 (8)
O4	0.1328 (18)	0.0430 (11)	0.0518 (11)	−0.0288 (11)	−0.0176 (11)	−0.0010 (9)
N1	0.0584 (14)	0.0572 (14)	0.0453 (13)	−0.0237 (11)	0.0023 (11)	−0.0054 (11)
N2	0.0468 (12)	0.0626 (14)	0.0431 (12)	−0.0214 (11)	0.0000 (10)	−0.0112 (10)
N3	0.0716 (15)	0.0397 (13)	0.0409 (12)	−0.0077 (11)	−0.0081 (10)	−0.0055 (10)
N4	0.0666 (14)	0.0372 (12)	0.0385 (11)	−0.0162 (10)	−0.0079 (9)	−0.0024 (9)
C1	0.0450 (13)	0.0445 (14)	0.0455 (14)	−0.0027 (11)	−0.0034 (11)	−0.0053 (11)
C2	0.0562 (15)	0.0556 (16)	0.0514 (15)	−0.0127 (13)	−0.0023 (12)	−0.0093 (12)
C3	0.0698 (18)	0.0677 (18)	0.0579 (17)	−0.0074 (15)	−0.0134 (14)	−0.0177 (15)
C4	0.076 (2)	0.078 (2)	0.0507 (17)	0.0048 (17)	−0.0169 (15)	−0.0081 (16)
C5	0.087 (2)	0.080 (2)	0.0591 (19)	−0.0181 (18)	−0.0171 (16)	0.0192 (16)
C6	0.0748 (19)	0.0598 (17)	0.0612 (18)	−0.0203 (15)	−0.0184 (15)	0.0077 (14)
C7	0.0483 (14)	0.0492 (15)	0.0496 (15)	−0.0124 (12)	−0.0012 (12)	−0.0062 (12)
C8	0.0461 (13)	0.0507 (15)	0.0486 (15)	−0.0121 (11)	0.0023 (11)	−0.0135 (12)
C9	0.0498 (15)	0.0667 (17)	0.0491 (15)	−0.0132 (13)	−0.0048 (12)	−0.0167 (13)
C10	0.0562 (15)	0.0521 (15)	0.0451 (14)	−0.0026 (12)	−0.0084 (12)	−0.0056 (12)
C11	0.0639 (16)	0.0404 (14)	0.0514 (15)	−0.0188 (12)	0.0041 (12)	0.0006 (11)
C12	0.0578 (15)	0.0444 (14)	0.0527 (15)	−0.0208 (12)	0.0023 (12)	−0.0082 (12)
C13	0.0527 (15)	0.0607 (16)	0.0485 (15)	−0.0100 (13)	−0.0065 (12)	−0.0144 (12)
C14	0.0612 (16)	0.0531 (15)	0.0379 (13)	−0.0040 (12)	−0.0102 (11)	−0.0100 (11)
C15	0.0512 (14)	0.0442 (14)	0.0399 (13)	−0.0115 (11)	−0.0051 (11)	−0.0044 (11)
C16	0.0543 (14)	0.0392 (14)	0.0426 (13)	−0.0097 (11)	−0.0041 (11)	−0.0023 (11)
C17	0.0440 (13)	0.0396 (13)	0.0399 (13)	−0.0052 (10)	−0.0027 (10)	−0.0009 (10)
C18	0.0591 (16)	0.0488 (15)	0.0492 (15)	−0.0153 (12)	−0.0033 (12)	0.0029 (12)
C19	0.0667 (18)	0.0656 (18)	0.0458 (16)	−0.0163 (15)	−0.0043 (13)	0.0132 (13)
C20	0.0631 (17)	0.080 (2)	0.0399 (14)	−0.0112 (15)	−0.0098 (13)	0.0033 (14)
C21	0.0691 (17)	0.0629 (17)	0.0527 (16)	−0.0161 (14)	−0.0134 (13)	−0.0059 (13)
C22	0.0572 (15)	0.0500 (15)	0.0434 (14)	−0.0135 (12)	−0.0065 (12)	0.0052 (11)

### Geometric parameters (Å, °)

**Table:**

S1—C8	1.665 (3)	C5—H5	0.9300
S2—C15	1.659 (2)	C6—H6	0.9300
O1—C7	1.220 (3)	C9—C10	1.492 (3)
O2—C10	1.417 (3)	C9—H9A	0.9700
O2—C11	1.424 (3)	C9—H9B	0.9700
O3—C12	1.419 (3)	C10—H10A	0.9700
O3—C13	1.419 (3)	C10—H10B	0.9700
O4—C16	1.222 (3)	C11—C12	1.485 (3)
N1—C7	1.366 (3)	C11—H11A	0.9700
N1—C8	1.396 (3)	C11—H11B	0.9700
N1—H1	0.79 (3)	C12—H12A	0.9700
N2—C8	1.311 (3)	C12—H12B	0.9700
N2—C9	1.446 (3)	C13—C14	1.501 (3)
N2—H2	0.82 (3)	C13—H13A	0.9700
N3—C15	1.310 (3)	C13—H13B	0.9700
N3—C14	1.451 (3)	C14—H14A	0.9700
N3—H3	0.83 (3)	C14—H14B	0.9700
N4—C16	1.367 (3)	C16—C17	1.486 (3)
N4—C15	1.404 (3)	C17—C22	1.382 (3)
N4—H4	0.82 (3)	C17—C18	1.386 (3)
C1—C6	1.382 (3)	C18—C19	1.372 (4)
C1—C2	1.387 (3)	C18—H18	0.9300
C1—C7	1.486 (3)	C19—C20	1.369 (4)
C2—C3	1.375 (4)	C19—H19	0.9300
C2—H2A	0.9300	C20—C21	1.380 (4)
C3—C4	1.376 (4)	C20—H20	0.9300
C3—H3A	0.9300	C21—C22	1.386 (4)
C4—C5	1.366 (4)	C21—H21	0.9300
C4—H4A	0.9300	C22—H22	0.9300
C5—C6	1.381 (4)		
			
C10—O2—C11	113.12 (18)	H10A—C10—H10B	108.5
C12—O3—C13	112.15 (18)	O2—C11—C12	108.43 (19)
C7—N1—C8	129.4 (2)	O2—C11—H11A	110.0
C7—N1—H1	117.6 (19)	C12—C11—H11A	110.0
C8—N1—H1	112.9 (19)	O2—C11—H11B	110.0
C8—N2—C9	124.1 (2)	C12—C11—H11B	110.0
C8—N2—H2	117.9 (18)	H11A—C11—H11B	108.4
C9—N2—H2	117.9 (19)	O3—C12—C11	109.5 (2)
C15—N3—C14	122.6 (2)	O3—C12—H12A	109.8
C15—N3—H3	119 (2)	C11—C12—H12A	109.8
C14—N3—H3	118 (2)	O3—C12—H12B	109.8
C16—N4—C15	127.9 (2)	C11—C12—H12B	109.8
C16—N4—H4	118.1 (18)	H12A—C12—H12B	108.2
C15—N4—H4	113.8 (18)	O3—C13—C14	108.6 (2)
C6—C1—C2	118.7 (2)	O3—C13—H13A	110.0
C6—C1—C7	123.7 (2)	C14—C13—H13A	110.0
C2—C1—C7	117.6 (2)	O3—C13—H13B	110.0
C3—C2—C1	120.8 (3)	C14—C13—H13B	110.0
C3—C2—H2A	119.6	H13A—C13—H13B	108.4
C1—C2—H2A	119.6	N3—C14—C13	111.0 (2)
C2—C3—C4	119.8 (3)	N3—C14—H14A	109.4
C2—C3—H3A	120.1	C13—C14—H14A	109.4
C4—C3—H3A	120.1	N3—C14—H14B	109.4
C5—C4—C3	119.9 (3)	C13—C14—H14B	109.4
C5—C4—H4A	120.0	H14A—C14—H14B	108.0
C3—C4—H4A	120.0	N3—C15—N4	116.7 (2)
C4—C5—C6	120.5 (3)	N3—C15—S2	124.06 (19)
C4—C5—H5	119.7	N4—C15—S2	119.18 (18)
C6—C5—H5	119.7	O4—C16—N4	121.1 (2)
C5—C6—C1	120.2 (3)	O4—C16—C17	121.1 (2)
C5—C6—H6	119.9	N4—C16—C17	117.8 (2)
C1—C6—H6	119.9	C22—C17—C18	119.0 (2)
O1—C7—N1	121.3 (2)	C22—C17—C16	124.1 (2)
O1—C7—C1	121.7 (2)	C18—C17—C16	116.9 (2)
N1—C7—C1	117.0 (2)	C19—C18—C17	120.5 (3)
N2—C8—N1	116.1 (2)	C19—C18—H18	119.8
N2—C8—S1	125.2 (2)	C17—C18—H18	119.8
N1—C8—S1	118.66 (18)	C20—C19—C18	120.4 (2)
N2—C9—C10	110.6 (2)	C20—C19—H19	119.8
N2—C9—H9A	109.5	C18—C19—H19	119.8
C10—C9—H9A	109.5	C19—C20—C21	120.1 (3)
N2—C9—H9B	109.5	C19—C20—H20	120.0
C10—C9—H9B	109.5	C21—C20—H20	120.0
H9A—C9—H9B	108.1	C20—C21—C22	119.7 (3)
O2—C10—C9	107.14 (19)	C20—C21—H21	120.1
O2—C10—H10A	110.3	C22—C21—H21	120.1
C9—C10—H10A	110.3	C17—C22—C21	120.3 (2)
O2—C10—H10B	110.3	C17—C22—H22	119.8
C9—C10—H10B	110.3	C21—C22—H22	119.8
			
O2—C11—C12—O3	65.0 (2)		

### Hydrogen-bond geometry (Å, °)

**Table:**

*D*—H···*A*	*D*—H	H···*A*	*D*···*A*	*D*—H···*A*
N2—H2···O1	0.82 (3)	1.99 (3)	2.648 (3)	136 (2)
N3—H3···O4	0.83 (3)	2.01 (3)	2.632 (3)	132 (3)
N4—H4···O2^i^	0.82 (3)	2.48 (3)	3.290 (3)	170 (2)

Symmetry codes: (i) −*x*+2, −*y*, −*z*+1.
